# Albendazole induces oxidative stress and DNA damage in the parasitic protozoan *Giardia duodenalis*

**DOI:** 10.3389/fmicb.2015.00800

**Published:** 2015-08-06

**Authors:** Rodrigo Martínez-Espinosa, Raúl Argüello-García, Emma Saavedra, Guadalupe Ortega-Pierres

**Affiliations:** ^1^Departamento de Genética y Biología Molecular, Centro de Investigación y de Estudios Avanzados del Instituto Politécnico NacionalMéxico City, Mexico; ^2^Department of Biochemistry, Instituto Nacional de Cardiología Ignacio ChávezMéxico City, Mexico

**Keywords:** *Giardia duodenalis*, albendazole, oxidative stress, DNA damage, apoptosis

## Abstract

The control of *Giardia duodenalis* infections is carried out mainly by drugs, among these albendazole (ABZ) is commonly used. Although the cytotoxic effect of ABZ usually involves binding to β-tubulin, it has been suggested that oxidative stress may also play a role in its parasiticidal mechanism. In this work the effect of ABZ in *Giardia* clones that are susceptible or resistant to different concentrations (1.35, 8, and 250 μM) of this drug was analyzed. Reactive oxygen species (ROS) were induced by ABZ in susceptible clones and this was associated with a decrease in growth that was alleviated by cysteine supplementation. Remarkably, ABZ-resistant clones exhibited partial cross-resistance to H_2_O_2,_ whereas a *Giardia* H_2_O_2_-resistant strain can grow in the presence of ABZ. Lipid oxidation and protein carbonylation in ABZ-treated parasites did not show significant differences as compared to untreated parasites; however, ABZ induced the formation of 8OHdG adducts and DNA degradation, indicating nucleic acid oxidative damage. This was supported by observations of histone H2AX phosphorylation in ABZ-susceptible trophozoites treated with 250 μM ABZ. Flow cytometry analysis showed that ABZ partially arrested cell cycle in drug-susceptible clones at G2/M phase at the expense of cells in G1 phase. Also, ABZ treatment resulted in phosphatidylserine exposure on the parasite surface, an event related to apoptosis. All together these data suggest that ROS induced by ABZ affect *Giardia* genetic material through oxidative stress mechanisms and subsequent induction of apoptotic-like events.

## Introduction

*Giardia duodenalis* is an intestinal parasitic protozoan that causes the infection known as giardiasis which affects about 280 million people around the world; 500,000 new cases are reported each year (Lane and Lloyd, 2002; Plutzer et al., 2010). This parasite is orally transmitted by the ingestion of infective cysts. Once in the host’s stomach excystation occurs and trophozoites emerge. At the duodenum the parasites replicate and colonize this portion of the intestine. Subsequently trophozoites are transported by peristalsis to the jejunum and the ileum where encystation takes place and mature cysts are expelled in the stool (Ankarklev et al., 2010; Watkins and Eckmann, 2014). The infection can be asymptomatic or present several clinical manifestations ranging from mild to severe symptoms that include diarrhea, steatorrhea, post-prandial epigastric pain, anorexia, bloating, and flatulence (Rossignol et al., 2012; Nash, 2013). Some infected patients may develop a chronic infection with recurrent diarrhea, steatorrhea, malabsorption, weight loss, and poor growth in children (Plutzer et al., 2010; Watkins and Eckmann, 2014).

The control of this infection is mainly carried out by treatment with chemotherapeutic agents. Among the drugs used are components that belong to 5-nitroimidazoles (e.g., metronidazole) and benzimidazoles (e.g., albendazole) derivatives. Other drugs prescribed against *Giardia* include nitazoxanide, furazolidone, paromomycin, and quinacrine (Tejman-Yarden and Eckmann, 2011; Watkins and Eckmann, 2014). Among these drugs albendazole (ABZ) is given in massive chemotherapy interventions against helminths based on its relative safety, high efficacy, broad spectrum against helminths, and low cost (Rossignol, 2010; Watkins and Eckmann, 2014). Further ABZ has been used against *Giardia*, particularly when metronidazole refractory cases occur (Lemée et al., 2000; Solaymani-Mohammadi et al., 2010). The side effects of this drug are rare but in some cases anorexia, constipation and neutropenia have been reported (Dayan, 2003). The use of ABZ is contraindicated during pregnancy due to possible teratogenic effects, although such effects have not been entirely confirmed (Gardner and Hill, 2001; Dayan, 2003).

In pharmacokinetic studies it has been determined that after its absorption, ABZ is oxidized to its metabolites, sulphoxide and sulphone, by cytochrome P450 and/or by flavin-dependent oxidases (Dayan, 2003). The production of these metabolites has been reported in *Giardia* after ABZ exposure (Oxberry et al., 2000; Argüello-García et al., 2015). It has also been reported that in helminths and fungi ABZ selectively binds to four β-tubulin sites, preventing its polymerization and affecting microtubule stability which in turn inhibits mobility and transport of molecules within the microorganism (Robinson et al., 2004; Diawara et al., 2013; Watkins and Eckmann, 2014). In helminths, ABZ-resistant parasites harbor *hot-spot* mutations in β-tubulin encoding different amino acids, particularly at glutamate 198 and phenylalanine 200 (Rossignol, 2010; Diawara et al., 2013; Hansen et al., 2013). In *Giardia*, it has been established that *hot-spot* amino acid mutations in β-tubulin are absent (Upcroft et al., 1996; Argüello-García et al., 2009) suggesting that the induction of ABZ-resistant phenotypes involves different mechanisms.

Regarding ABZ resistance in *Giardia*, it has been reported that resistant trophozoites display morphological changes, particularly in the median body, despite the conserved amino acid residues at positions 198 and 200 in β-tubulin (Chavez et al., 1992; Upcroft et al., 1996; Argüello-García et al., 2009). On the other hand, chromosomal rearrangements have been documented in ABZ-treated parasites, although there is no evidence of a gene or group of genes that may be affected during ABZ resistance in this parasite (Upcroft and Upcroft, 2001). Previous studies by our group suggest that diverse metabolic mechanisms may be involved in the ABZ resistance in *Giardia* that could include components of antioxidant and energy metabolism as well as cytoskeletal changes in the parasite (Paz-Maldonado et al., 2013).

In this context, recent reports have suggested a direct relationship between the use of ABZ and oxidative stress. In a report in which ABZ was administered to rats in various doses and times, oxidative stress was elicited particularly in hepatocytes (Locatelli et al., 2004). Other studies have also shown the ability of ABZ to induce oxidative stress in sheep liver (Dimitrijević et al., 2012), and ABZ consumption may be correlated with liver damage in humans (Nandi and Sarkar, 2013). In *Dicrocoelium dendriticum*, a fluke of veterinary and human health importance, an increase in antioxidant enzyme activity after ABZ exposure was identified (Bártíková et al., 2010). However, no reports on oxidative damage due to ABZ in other parasites are available.

To determine in more detail the mode of action of ABZ in *Giardia*, in this work we have assessed the induction of oxidative stress by ABZ in *G. duodenalis* trophozoites by monitoring reactive oxygen species (ROS) formation. Results identified ABZ-induced oxidative stress in this protozoan. Oxidative damage to the parasite´s DNA is associated with cell cycle arrest and apoptosis. The consequences of this stress and its possible relationship to ABZ resistance in *Giardia* are discussed.

## Materials and Methods

### Trophozoite Cultures, Growth of ABZ-Resistant Clones and Obtention of H_2_O_2_-Resistant Trophozoites

*Giardia duodenalis* trophozoites of the WB strain (ATCC#30957) and ABZ-resistant clones were maintained in TYI-S-33 medium supplemented with 10% adult bovine serum (HyClone) and antibiotic/antimycotic solution (Thermo, USA) at 37°C (Keister, 1983) in 4.5 mL screw-capped vials. ABZ-resistant trophozoites were selected by continuous subculture under increasing sub-lethal concentrations of ABZ (Sigma cat. A-4673). When parasites were adapted to each increase of drug concentration, cultures were cloned by limiting dilution using the corresponding ABZ concentration (Paz-Maldonado et al., 2013). Trophozoites were sub-cultured twice a week under the continuous presence of drug (for ABZ-resistant clones) and for the ABZ-sensitive clones only in the presence of the vehicle (N, N-dimethylformamide; DMF, Sigma). To obtain the H_2_O_2_-resistant parasites (ROX), trophozoites were selected by continuous subculture under increasing sub-lethal concentrations of H_2_O_2_ (Sigma, USA). Vials containing trophozoites were refilled to three-quarter capacity and H_2_O_2_-resistant parasites were cultured as described above for ABZ-resistant *Giardia*. Stock solutions (0.01–25 mM) of ABZ in DMF or DMF alone were used in all assays. Oxidative stress was induced by exposing the parasites to 100 μM H_2_O_2_ and these cultures were used as positive controls (Raj et al., 2013).

### Determination of Trophozoite Growth

Trophozoite growth was assessed by the fluorescent tracer SYTOX Green according to the manufacturer’s instructions (Invitrogen, USA). Briefly, trophozoites were washed three times in phosphate buffered saline (PBS) then suspended in lysis buffer (6% SDS, 10 mM HEPES) using a Vortex shaker for 10 s. A stock solution of SYTOX Green (5 mM) was added in a 1:5 v/v ratio and incubated for 10 min in the dark. The standard growth curve was obtained using variable numbers of lysed trophozoites and absorbance values of each sample from non-treated and treated trophozoites were determined in 96-well, black-bottomed microtiter plates using a FACSCalibur reader fitted with 504/525 nm excitation/emission filters (Gerphagnon et al., 2013). Negative control absorbance values were obtained from wells with no cells.

### Detection of Reactive Oxigen Species (ROS) in Trophozoites Incubated with ABZ or H_2_O_2_

Albendazole-sensitive trophozoites were incubated with ABZ (1.35, 8, and 250 μM), Dimethylformamide (DMF referred as vehicle) or H_2_O_2_ (100 μM) as control for oxidative stress, for 16 h at 37°C. ROS formation was assessed by Image-IT LIVE Green Reactive Oxygen Species Detection Kit^TM^ according to manufacturer´s instructions (Life Technologies, USA). After incubation, trophozoites were washed in PBS and suspended in 25 μM 6-carboxy-2′,7′-dichlorodihydrofluorescein diacetate (carboxy- H2DCFDA) at 37°C for 30 min. Then Hoechst 33342 was added at a final concentration of 1 μM for 5 min. Cell fluorescence signals were detected at the end of the incubation period in a Beckman FACSCalibur Flow Cytometer or in an optical microscope using the BD FACSComp software.

### Determination of Cross-Resistance to ABZ and H_2_O_2_ and Protection by Cysteine

The cross resistance between ABZ and H_2_O_2_ was evaluated using the ABZ-resistant and H_2_O_2_-resistant clones mentioned above. The ABZ-resistant clones (R1.35, R8, R250) were exposed to 0, 25, 50, 75, and 100 μM H_2_O_2_ for 24 h at 37°C. Cell number was determined by SYTOX Green. Resistance to ABZ was determined in ROX (H_2_O_2_-resistant) parasites which were exposed to 0.05, 0.1, 0.2, 0.4, and 0.8 μM of ABZ for 24 h at 37°C. In control cultures, ABZ or H_2_O_2_ were not added. Cell number was also determined using SYTOX Green. For cysteine protection assays, ABZ-sensitive trophozoites were grown in TYI-S-33 medium supplemented with different concentrations of cysteine (0.5, 1, 2, or 4 mM). Then, trophozoites were incubated in the presence of 0.2 μM ABZ for 48 h at 37°C. Cell growth was determined by SYTOX Green as indicated above.

### Detection of Protein Carbonylation and Lipid Peroxidation

Albendazole-sensitive trophozoites were incubated with DMF, ABZ (1.35, 8, and 250 μM) or H_2_O_2_ (100 μM) for 24 h at 37°C. Protein carbonylation was determined using a commercial kit (Protein Carbonyl Assay, Cayman Chemical, USA). Trophozoites were washed with PBS, suspended in lysis solution (50 mM MES, 1 mM EDTA at pH 7.4) and lysed by three cycles of freezing-thawing followed by centrifugation at 10,000 x *g* for 10 min. Subsequently the protein was derivatized with dinitrophenylhydrazine (DNPH) for 60 min in the dark, the reaction was stopped with 20% trichloroacetic acid and samples were centrifuged at 10,000 × *g* for 10 min. Then the samples were washed three times with ethanol/ethyl acetate solution. Finally, the proteins were suspended in guanidine hydrochloride and the absorbance was determined at 450 nm (Krisko and Radman, 2010). The concentration of protein in the soluble fraction was determined by absorbance at 280 nm.

For lipid peroxidation determination, after incubation with compounds or vehicle a solution of 1-methyl-2-phenylindole in a mixture of acetonitrile/methanol (3:1) was added to trophozoite homogenates. For malondialdehyde (MDA) determination, the reaction was initiated by adding HCl to a 37% v/v final concentration and for the 4-hydroxynonenal (HNE) assay methanesulfonic acid and FeCl_3_ at 34 μM (final concentration each) were used. The absorbance at 586 nm was measured upon incubation of the reaction mixture at 45°C for 40 min. For each series of assays, the absorbance of a control containing water instead of a sample was always subtracted. For each assay homogenate, a control sample in which the reagent was replaced by acetonitrile/methanol (3:1, v/v) was included. A standard curve of trimethoxypropane was used in all assays (Gérard-Monnier et al., 1998; Orozco-Ibarra et al., 2007).

### Detection of DNA Fragmentation

Albendazole-sensitive trophozoites were incubated with different concentrations of ABZ (1.35, 8 and 250 μM), DMF or H_2_O_2_ (100 μM) for 24 h at 37°C, then washed twice in PBS 1X and incubated overnight at 42°C in a lysis solution (10 mM Tris-HCl, pH 7.4, 10 mM EDTA, 150 mM NaCl, 0.4% sodium dodecyl sulfate, and 200 μg/mL proteinase K). RNA was removed by incubating samples with 20 mg/mL RNase A at 37°C for 30 min. The lysate was treated with phenol/chloroform (1:1) and nucleic acids were precipitated at -20°C with 0.3 M sodium acetate pH 7 and ethanol. After quantification, the extent of DNA fragmentation was analyzed by electrophoresis on 1% agarose/ethidium bromide gels (Ghosh et al., 2009).

### Detection of Oxidative DNA Damage

DNA damage was assessed by immunofluorescence with an anti-8-hydroxydeoxyguanosine (8OHdG) monoclonal antibody (Santa Cruz Technologies, USA). ABZ-sensitive trophozoites treated with DMF, different concentrations of ABZ (1.35, 8, and 250 μM) or H_2_O_2_ (100 μM) for 16 h at 37°C were incubated for 1 h at 37°C on poly-L-lysine-coated (2 mg/ml) coverslips, rinsed twice with PBS and fixed with a solution of methanol:acetone (1:1 v/v) at -20°C. Fixed cells were treated with 0.05 N HCl for 5 min on ice, rinsed with PBS and washed with PBS containing 35, 50, and 75% ethanol consecutively for 3 min each time. DNA was denatured *in situ* with 0.15 N NaOH in 70% ethanol for 4 min. The precipitate was rinsed twice with PBS and incubated with 0.2 μg/ml Hoechst dye for 10 min. Subsequently parasites were washed with PBS containing 75, 50, and 35% ethanol consecutively in the presence of 4% formaldehyde for 2 min each time. The samples were incubated in trypsin solution (49.5 mM Tris base, 1 mM EDTA, 150.7 mM Na_2_HPO_4_, 14.9 mM K_2_HPO_4_, 0.1% trypsin at pH 7.2) for 10 min at 37°C and washed three times with PBS. Trophozoites were then incubated for 30 min with 1% bovine serum albumin (BSA) to block nonspecific binding and incubated with mouse monoclonal anti- 8-OHdG for 1 h. After a wash with PBS, cells were incubated for 1 h at room temperature with goat anti-mouse IgG coupled to FITC (Santa Cruz Technologies, USA). Samples were analyzed using a Zeiss microscope equipped with epifluorescence illumination as previously described (Suzuki et al., 2006).

### Detection of Protein-MDA Adducts and H2AX Phosphorylation by Western Blot

Both protein-MDA adducts and histone H2AX phosphorylation (at ser139) were evaluated by Western blot assays using specific antibodies. In these, ABZ-sensitive trophozoites were incubated with DMF, different concentrations of ABZ (1.35, 8, and 250 μM) or H_2_O_2_ (100 μM) for 24 h at 37°C, washed with PBS tree times and suspended in lysis buffer. Twenty microgram of protein were analyzed by SDS-PAGE in 12% acrylamide gels for the protein-MDA assay and in 15% acrylamide gels for detection of H2AX phosphorylation. After electrophoresis, gels were transferred to nitrocellulose membranes. Membranes were blocked with PBS containing 0.1% Tween-20 and 1% skim milk for 2 h at 37°C. After washing with Tris-buffered saline (TBS) membranes were incubated with rabbit anti-MDA (Abcam, USA) and rabbit anti-H2AX (Millipore, USA) antibodies for 1 h at room temperature under constant shaking. Membranes were washed and incubated with horseradish peroxidase-conjugated mouse anti-rabbit IgG (Thermo, USA). Chemiluminescence detection was performed with the Amersham ECL detection kit according to manufacturer’s instructions (Hofštetrová et al., 2010; Moore et al., 2013).

### Identification and Quantification of Apoptotic and Necrotic Cells

The cells undergoing apoptotic or necrotic processes after ABZ- or H_2_O_2_ exposure were analyzed by flow cytometry in which fluorescence by annexin V binding (green) and propidium iodide (PI) uptake (red) were quantified. Positioning of quadrants on annexin V/PI dot plots was analyzed according to the following pattern: living cells (annexin V-/PI-), early apoptotic/primary apoptotic cells (annexin V+/PI-), late apoptotic/secondary apoptotic cells (annexin V+/PI+) and necrotic cells (annexin V-/PI+). The assay was carried out using the Annexin V-FITC Apoptosis Detection Kit (BioVision, USA) following the manufacturer´s instructions. Briefly, cells were incubated with DMF, different concentrations of ABZ (1.35, 8, and 250 μM) or H_2_O_2_ (100 μM) for 24 h. Then, trophozoites were centrifuged at 440 × *g* at 4°C and suspended in 500 μl of 1X binding buffer. Cells were then incubated with 5 μl of annexin V–FITC and 5 μl of PI (50 μg/ml) for 5 min in the dark at room temperature. The FITC and PI fluorescence was measured with a FACS Calibur Flow Cytometer equipped with an FL-1 filter (530 nm) and an FL-2 filter (585 nm), respectively, in at least 10,000 events (Ghosh et al., 2009) in each experiment.

### Determination of Cell Cycle Stages in *G. duodenalis* Trophozoites Exposed to ABZ

To determine the proportions of trophozoites at the different cycle stages, nuclear staining with PI was coupled to flow cytometry. In brief, ABZ-sensitive trophozoites were exposed to different concentrations of ABZ (1.35, 8, and 250 μM) for 4 h at 37°C, washed with PBS and fixed 30 min with 70% ethanol in PBS. Then cells were washed again and incubated in PBS containing 0.1 mg/mL RNAase overnight at 4°C. Finally cell pellets were washed, stained with PI (1 μM in PBS), washed and resuspended in small volume (200–300 μL) for analysis in a FACS Calibur Flow Cytometer in at least 10,000 events per sample. The histogram areas were identified as reported by Reaume et al. (2013).

### Statistical Analyses

All the data were obtained from at least three experiments and where indicated the results are expressed as mean ± SD. Inter-group variation was assessed by one-way analysis of variance (ANOVA) followed by Tukey’s multiple comparison test. Statistical significance was determined if *p* ≤ 0.05.

## Results

### Intracellular ROS Formation

**FIGURE 1:**
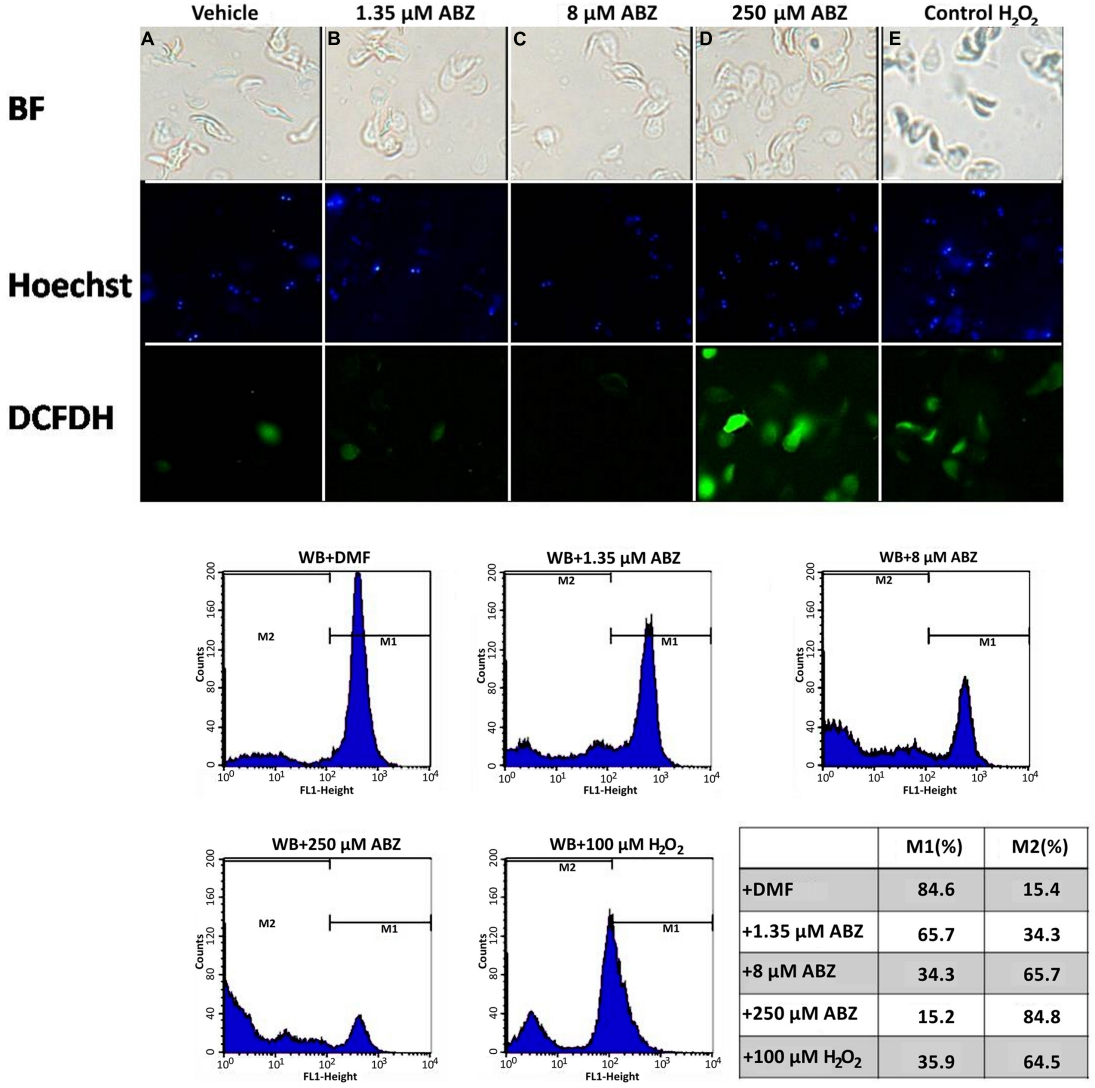
**Reactive oxygen species (ROS) are produced in *Giardia duodenalis* trophozoites exposed to Albendazole (ABZ).** ABZ-sensitive *Giardia* strain (WB) was exposed to vehicle (DMF, **A**) and to 1.35 μM **(B)**, 8 μM **(C)**, or 250 μM **(D)** of ABZ for 8 h at 37°C, then DCFDH was added to monitor ROS production. In these experiments a control of intracellular ROS production was included. In this WB trophozoites were incubated with 100 μM H_2_O_2_ **(E)**. Trophozoites micrographs are as follows: top panels bright field (BF), middle panel trophozoites’ nuclei stained with Hoechst and lower panel trophozoites stained with DCFDH. The cells showed increased ROS production by ABZ treatment in comparison to control cells with no drug. Bottom panels are ROS production monitored by flow cytometry, the shift of fluorescence in *X* axis indicates ROS production by live trophozoites (determined by Trypan blue exclusion) at the highest ABZ concentration. The table shows population percent in M1 (negative to ROS) and M2 (positive to ROS) according to flow cytometry data. Micrographs are from representative results of at least three experiments performed with independent batch cultures.

In some reports using animal models ABZ was shown to produce oxidative damage (Locatelli et al., 2004; Bártíková et al., 2010). In *G. duodenalis* oxidative stress damage has been induced using pro-oxidant compounds such as H_2_O_2_ (Ghosh et al., 2009; Raj et al., 2013) in which intracellular ROS formation hallmarks this phenomenon. ABZ-exposed parasites showed greater ROS signals than parasites not exposed to the drug (**Figure 1**, top panels). The effect was mainly detected at the highest ABZ concentration tested (250 μM), however, ROS formation could be determined by flow cytometry at lower concentrations (**Figure 1**, bottom panels).

**FIGURE 2:**
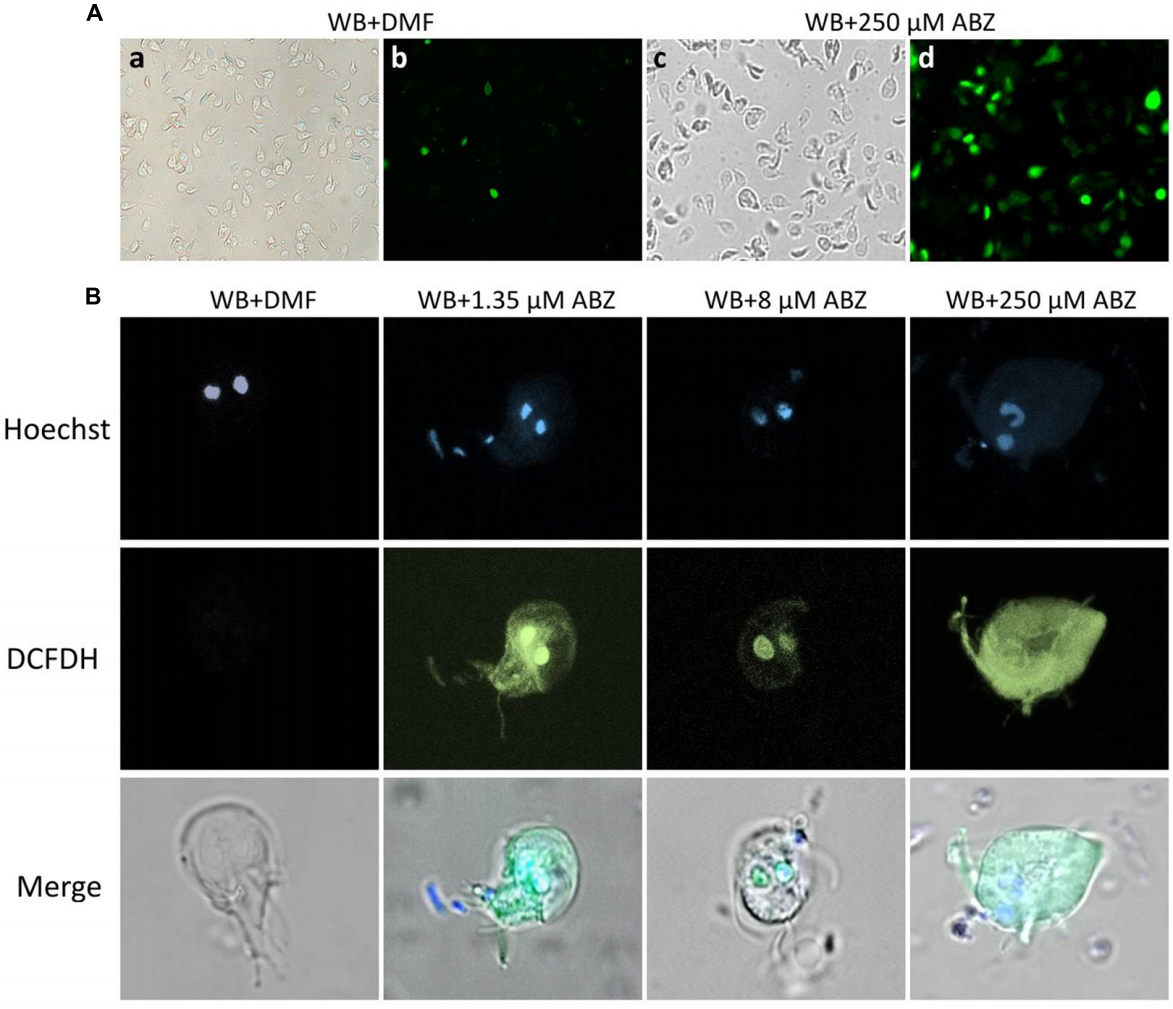
**Intracellular localization of ROS production in *G. dudodenalis* trophozoites exposed to ABZ.** WB *Giardia* trophozoites were exposed to DMF or to the indicated ABZ concentrations (from left to right: 1.35, 8, and 250 μM) for 8 h at 37°C. Cells were then incubated with DCFDH. Trophozoite micrographs are as follows: **(A)** top panel: representative images of trophozoites exposed to DMF (a,b) or to 250 μM ABZ (c,d) and then incubated with DCFDH. Morphological changes in trophozoites (c BF) and ROS localization (d epifluorescence illumination) are evident in ABZ treated cells. **(B)** Images of representative individual cells. Top panel trophozoites´ nuclei stained with Hoechst, middle panel trophozoites incubated with DCFDH (epifluorescence illumination) and lower panel merged cell images. At the lowest concentrations, ROS production is restricted to nuclei, whereas at the highest ABZ concentration, this is detected all over the cytoplasm. The micrographs are representative of at least three independent experiments.

To determine the localization of intracellular ROS formation within the trophozoites confocal microscopy was used. In these experiments the typical altered morphology caused by benzimidazoles (Paz-Maldonado et al., 2013) was observed in ABZ-treated trophozoites (**Figure 2A** top panel c). In these cells ROS formation was also evident in most cells as determined by fluorescent staining (**Figure 2A** top panel d). When individual cells were observed the trophozoites´nuclei were determined as the primary site of ROS formation (**Figure 2B**) as judged by fluorescent staining at low ABZ concentrations used (1.35 μM and 8 μM). At the highest drug concentration used (250 μM) there was a widespread distribution of ROS throughout the trophozoite cytoplasm (**Figure 2B** middle panel).

### Cross Resistance to ABZ and H_2_O_2_ in *Giardia* Trophozites

**FIGURE 3:**
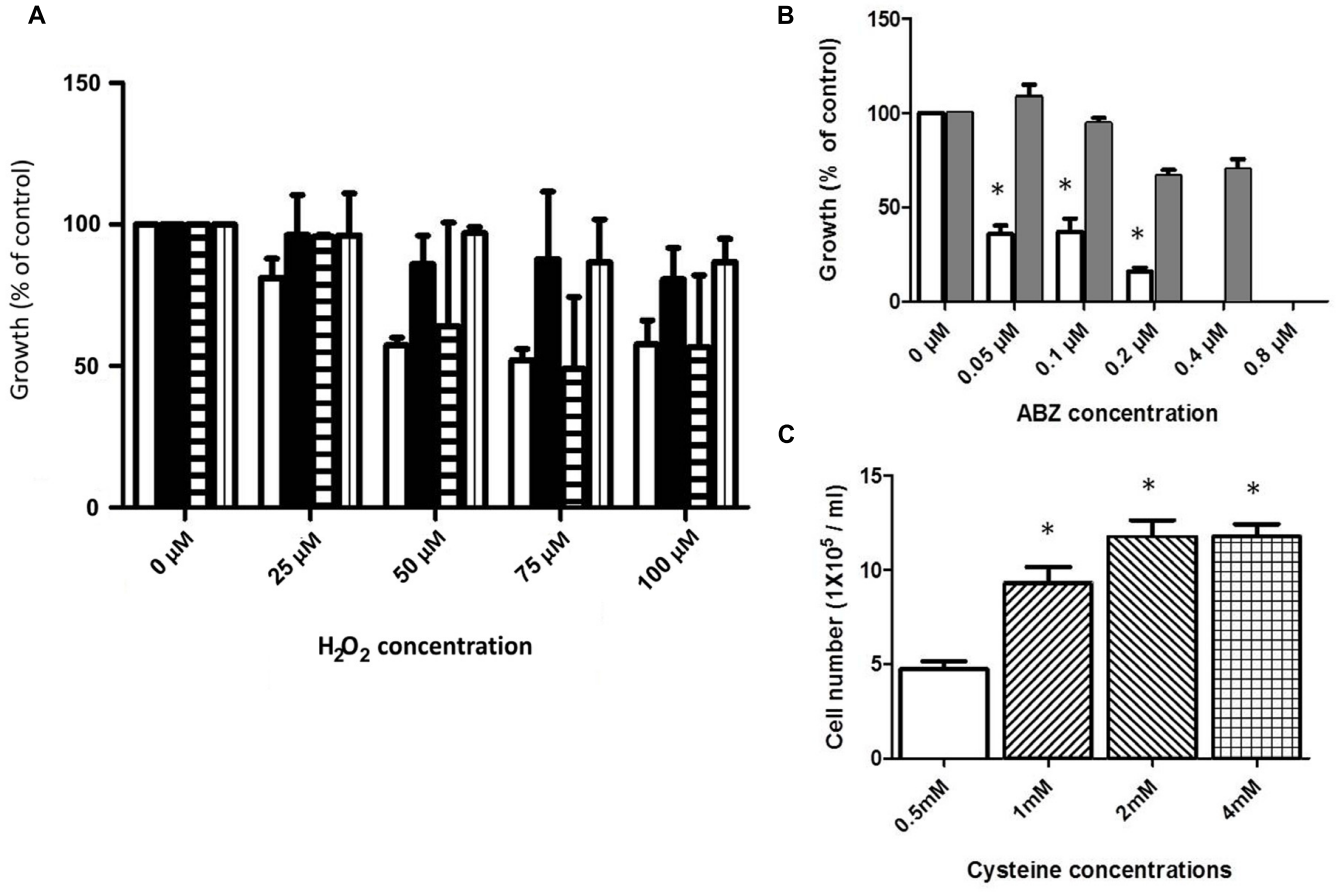
**Cross-resistance to ABZ and H_2_O_2_ and cysteine (Cys) protection in trophozoites exposed to ABZ. (A)** Cross-resistance to H_2_O_2_ was evaluated in the ABZ-resistant clones R1.35 (black bars), R.8 (horizontal lined bars), and R.250 (vertical lined bars) previously obtained in our group (Paz-Maldonado et al., 2013). Parasites were exposed to the indicated H_2_O_2_ concentrations for 24 h and the cell number was determined by SYTOX Green. As control, wild type WB strain (white bars) was incubated with the indicated H_2_O_2_ concentration. **(B)** Cross-resistance to ABZ in a H_2_O_2_-resistant *G. dudodenalis.* A H_2_O_2_-resistant Giardia strain (ROX) was obtained by sub-culturing the WB strain in increasing H_2_O_2_ concentration for 6 months. Control WB (white bars) and ROX (gray bars) trophozoites were exposed to the indicated increasing ABZ concentrations for 24 h and the cell number was determined using the fluorescent tracer SYTOX Green. The ROX strain showed cross-resistance to ABZ. **(C)** Cysteine protection of *Giardia* trophozoites exposed to ABZ. WB trophozoites were incubated in growth medium with 0.5 mM (white bar), 1 mM (lined up to the right bar), 2 mM (lined up to the left bar), or 4 mM (boxed bar) of cysteine for 24 h and then trophozoites were further incubated with 0.2 μM ABZ for 48 h. In all graphs the results are the mean ± SD of at least three independent experiments. In graphs **(B**,**C)** ^∗^ indicates *p* ≤ 0.05 by ANOVA and Tukey´s analysis in which values obtained with WB trophozoites were compared with values obtained in ROX trophozoites exposed to the different ABZ concentrations **(B)**. **(C)** Values obtained in WB trophozoites treated with 0.5 mM cysteine were compared to values obtained in WB trophozoites treated with different cysteine concentrations and then exposed to ABZ.

The ABZ-resistant clones, namely R1.35, R.8, and R.250 (Argüello-García et al., 2009; Paz-Maldonado et al., 2013) were used to determine whether cross-resistance to classical oxidative stressor (H_2_O_2_) and ABZ was induced in the resistant trophozoites. For this purpose the ABZ-resistant clones were incubated under increasing concentrations of H_2_O_2_, and cell growth was determined. In general the resistant clones R1.35 and R.250 showed a tendency to increased resistance to H_2_O_2_-induced death in comparison to the ABZ-susceptible WB strain (**Figure 3A**). A special case is the R8 resistant strain which frequently behave, in this and other studies, as a “transition state” between low and high ABZ resistance depending on the parameter that is evaluated (see also **Figure 6A**; Argüello-García et al., 2009; Paz-Maldonado et al., 2013)

Resistance to H_2_O_2_ was achieved by using continuous trophozoite subcultures under increasing concentrations of H_2_O_2_. Following this procedure for approximately 6 months, a strain displaying resistance to 75 μM H_2_O_2_ was obtained (ROX). This strain was incubated in the presence of ABZ showing a significantly higher growth rate as compared with the H_2_O_2_-susceptible WB strain (**Figure 3B**).

### Cysteine Increases Tolerance to ABZ in the Drug Treated Trophozoites

*Giardia duodenalis* possesses a bacterial-like antioxidant metabolism in which cysteine is the major antioxidant thiol instead of glutathione. To test if increased levels of extracellular cysteine may confer increased tolerance to the drug in ABZ-susceptible trophozoites, these clones were incubated in culture media containing different cysteine concentrations and subsequently ABZ at 0.2 μM was added and after 48 h-incubation trophozoite growth was measured. As a result, parasites incubated at cysteine concentrations higher than 1 mM showed higher growth after ABZ exposure than those incubated at lower concentrations of this amino acid (**Figure 3C**). These results suggest that cysteine may help to contend the ROS damage upon ABZ exposure.

### Damage to Biomolecules after ABZ Exposure in *G. duodenalis* Trophozoites

**FIGURE 4:**
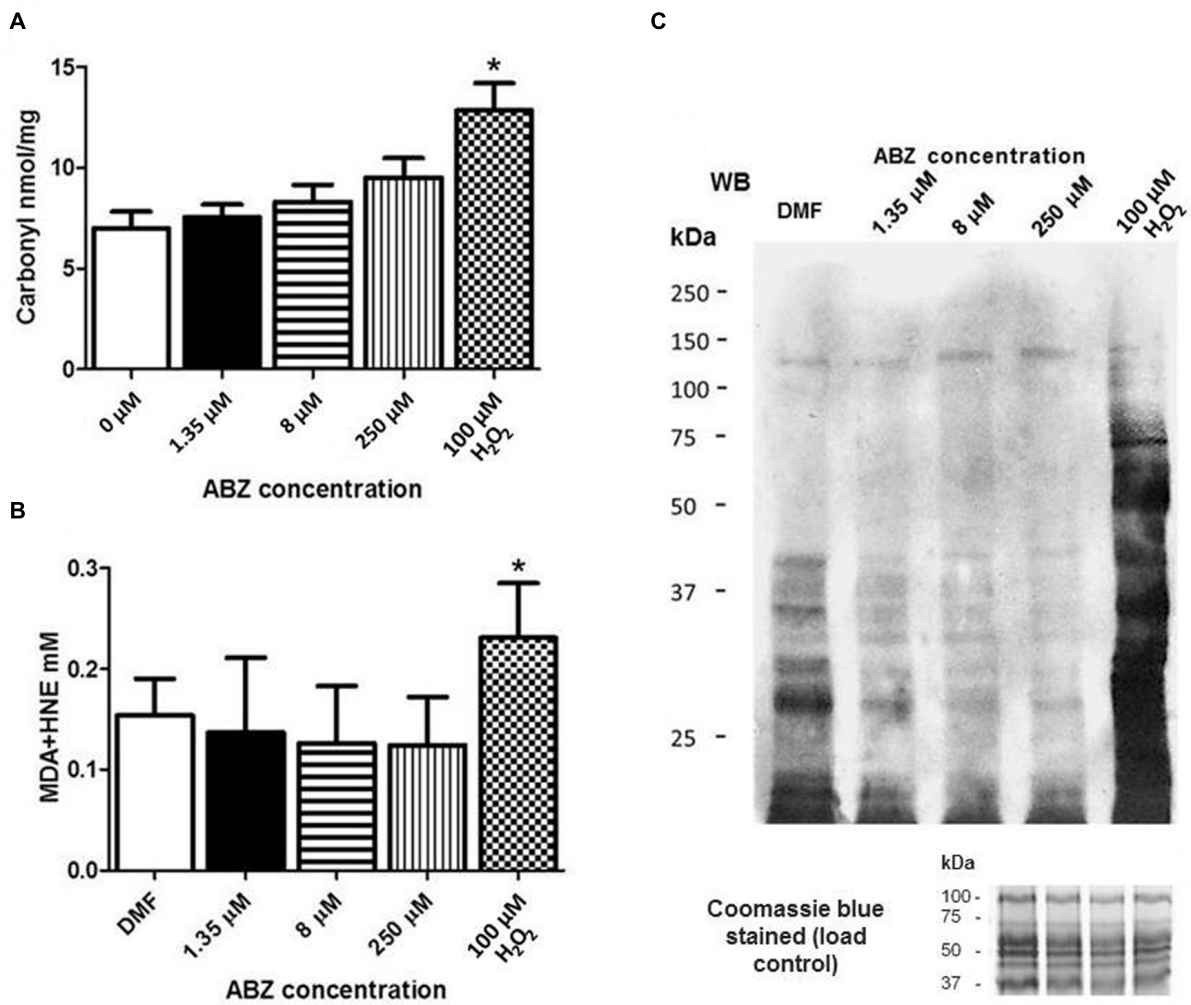
**Oxidative damage to proteins in *G. duodenalis* trophozoites exposed to ABZ. (A)** Protein carbonylation in *G. duodenalis* ABZ-sensitive trophozoites (WB) exposed to ABZ. Parasites were exposed to vehicle (DMF white bars) or to different ABZ concentrations (1.35 μM black bars; 8 μM horizontal lined bars; 250 μM vertical lined bars) for 16 h at 37°C. Control WB trophozoites were exposed to direct ROS damage with 100 μM H_2_O_2_ (white and black boxes). Proteins from each sample were obtained and were derivatized with DNPH for protein cabonylation determination. DNP-protein adducts were detected by absorbance at 450 nm. **(B)** Lipoperoxidation in trophozoites exposed to ABZ. After trophozoites were exposed to the different ABZ concentrations as indicated in **(A)**. HNE or MDA were determined by absorbance at 586 nm. The values in **(A,B)** are the mean ± SD. ^∗^*p* ≤ 0.05 by ANOVA and Tukey’s analysis in which values obtained with trophozoites treated with the different ABZ concentrations were compared to values obtained with the DMF treated parasites. **(C)** MDA-protein adducts were detected by Western blot in the same trophozoites samples treated with the indicated ABZ concentrations using rabbit anti-MDA antibodies. At the bottom of panel **(C)** the Coomassie blue stained gel is included and shows that similar protein amounts of each sample were loaded.

The formation of intracellular ROS may trigger oxidative damage to various biomolecules such as proteins, lipids, and DNA. Protein carbonylation was determined in ABZ-sensitive trophozoites exposed to increasing ABZ concentrations. As can be seen in **Figure 4A**, a consistent but not statistically significant increase in protein carbonylation correlated with the increase in drug concentration, confirming the presence of oxidative damage by ABZ treatment in cellular proteins of *G. duodenalis.*

The levels of lipid peroxidation in healthy cells are maintained under controlled limits but these can be affected, i.e., increased, when cells are treated with pro-oxidant xenobiotics. This process leads to the formation of some intermediates such as MDA and HNE. The determination of such intermediates was carried out after ABZ exposure of drug–sensitive trophozoites. In both cases there was not a significant change in ABZ-treated parasites as compared with untreated cultures contrary to the drug susceptible clones exposed to H_2_O_2_ used as a control (**Figure 4B**). This observation was further corroborated using antibodies detecting MDA-protein adducts which displayed more intense staining only when drug susceptible trophozoites were exposed to H_2_O_2_ (**Figure 4C**).

**FIGURE 5:**
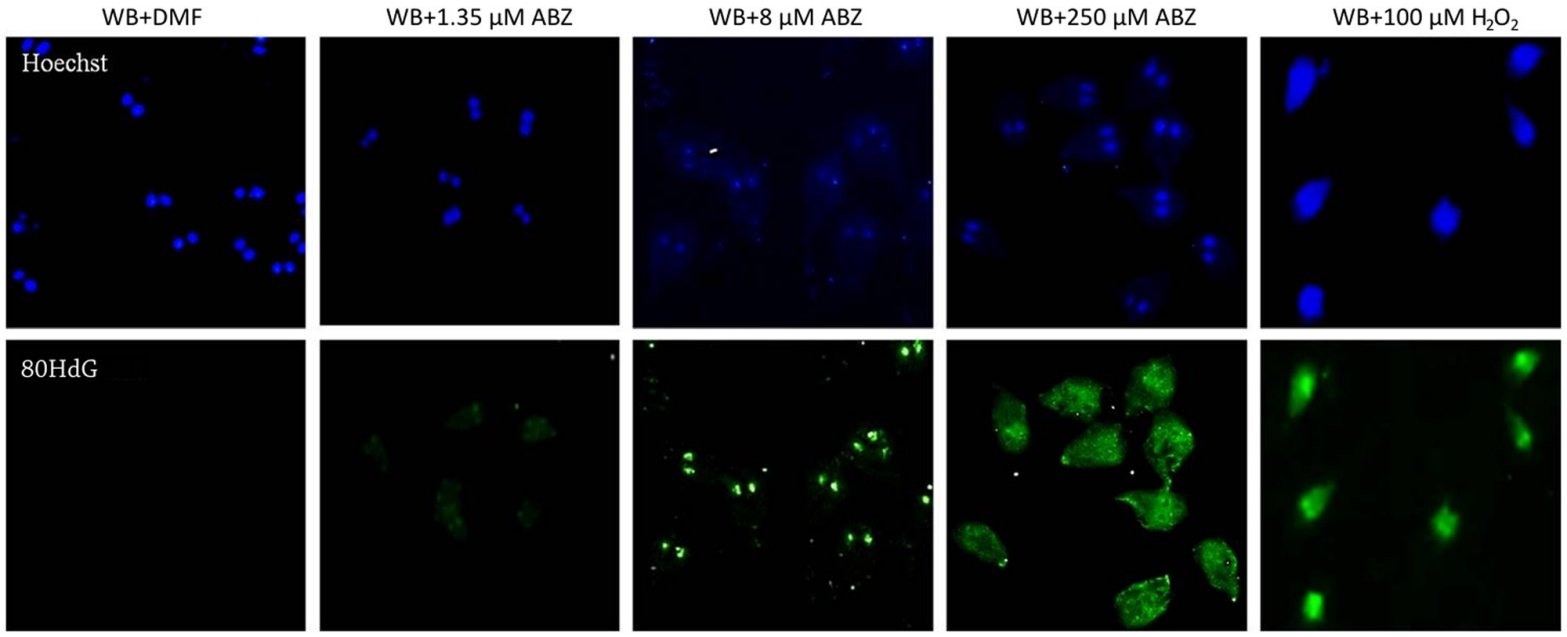
**Oxidative damage to DNA in *G. duodenalis* trophozoites exposed to ABZ.** Parasites of the WB strain were exposed to DMF, 1.35 μM, 8 μM, 250 μM of ABZ or 100 μM of H_2_O_2_ (the latter as control of ROS damage) for 8 h at 37°C. Then trophozoites were fixed on slides and incubated with the anti-8-hydroxydeoxyguanosine monoclonal antibody to detect modified nucleotides in DNA as described in materials and methods. Trophozoite micrographs are as follows: top panel trophozoites’ nuclei stained with Hoechst; lower panel reactivity of anti-8-hydroxydeoxyguanosine (8OHdG) antibodies in trophozoites’ nuclei (WB + 8 μM ABZ) and cytoplasm (WB + 250 μM ABZ). The figures are representative of at least three independent experiments.

Oxidative damage to DNA by ABZ treatment was determined by immunocytochemistry assays using antibodies against 8-OHdG. In these, a significant increase in intranuclear staining, denoting 8-OHdG-containing DNA in ABZ-treated cells was observed (**Figure 5**). At the highest drug concentrations used (250 μM) the fluorescent signal was observed in nucleus and the entire cytosol displayed a diffuse pattern with a punctuated pattern in some regions within the trophozoites (**Figure 5**).

### Characterization of DNA Damage in *G. duodenalis* Trophozoites Induced by ABZ

**FIGURE 6:**
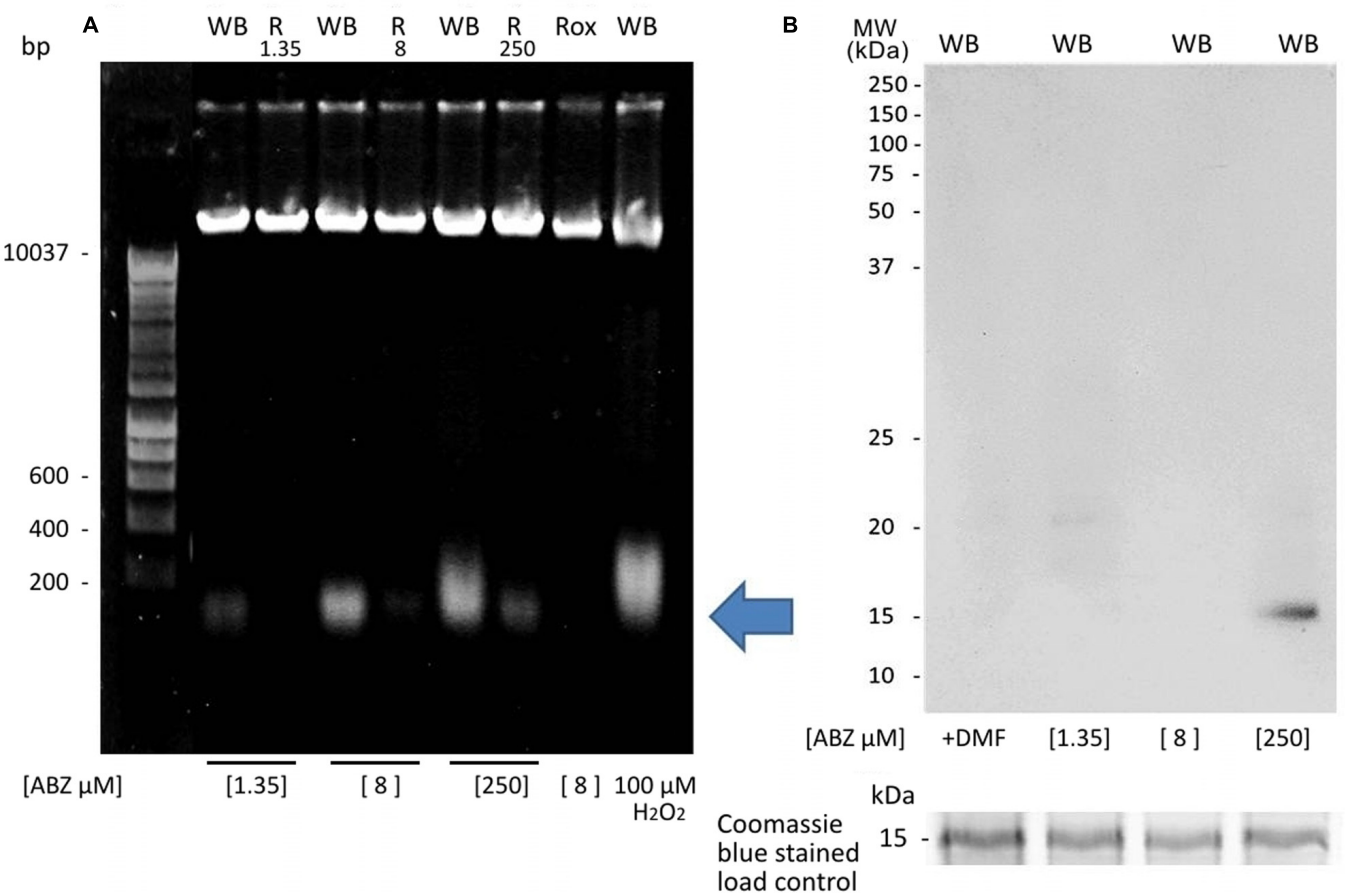
**DNA fragmentation and histone H2AX phosphorylation in *G. duodenalis* trophozoites exposed to ABZ. (A)** WB strain trophozoites (WB) were exposed to 1.35, 8, and 250 μM of ABZ or to 100 μM of H_2_O_2_ in parallel with the resistance clones (R1.35, R.8, R.250) and the H_2_O_2_-resistant strain (ROX) which was exposed only to 8 μM of ABZ. Then the cells were incubated for 16 h at 37°C. Total DNA was extracted, treated with RNAse and analyzed in agarose gels. DNA degradation zone is shown by an arrow. DNA degradation was mainly observed in the WB strain treated with the different concentrations of ABZ **(B)**. Histone H2AX phosphorylation was determined in total extract obtained from *G. duodenalis* WB trophozoites that were exposed to DMF and to different ABZ concentrations (from left to right: 1.35, 8, and 250 μM) for 16 h at 37°C. The reactivity of H2AX phosphorylation was determined by Western blot using rabbit anti-H2AX antibody. In the bottom of panel **(B)** the 15 kDa region of the Coomassie blue stained gel used as a load control is shown. The figures are representative of at least three independent experiments.

From the previous observations we concluded that ABZ caused a preferential oxidative damage at the DNA level over other biomolecules which could be severe as judged by the extra nuclear 8OHdG detection at higher ABZ concentrations. To assess damage to DNA integrity, genomic DNA was purified from control and ABZ-exposed trophozoites and analyzed in agarose gels to assess its integrity (presence of a high-sized band) or partial/total degradation. As shown in **Figure 6A**, genomic DNA was broken down in ABZ-exposed trophozoites as determined by the presence of diffuse and low molecular weight bands. This DNA degradation was absent or present at a lesser extent in the ABZ- and ROX-resistant strains. To further confirm the damage to the double stranded DNA, anti-histone H2AX antibodies were used. This protein becomes phosphorylated upon DNA double-strand break. In **Figure 6B** it is shown that exposure of parasites to the highest ABZ concentration (250 μM) induced a H2A signal indicating damage to DNA. The level of phosphorylated *Giardia* H2A was proportional to the DNA damage, therefore the signal was better observed at the highest concentration of ABZ.

### ABZ Induces Apoptosis-Like Death and Partial Cell Cycle Arrest in *G. duodenalis* Trophozoites

**FIGURE 7:**
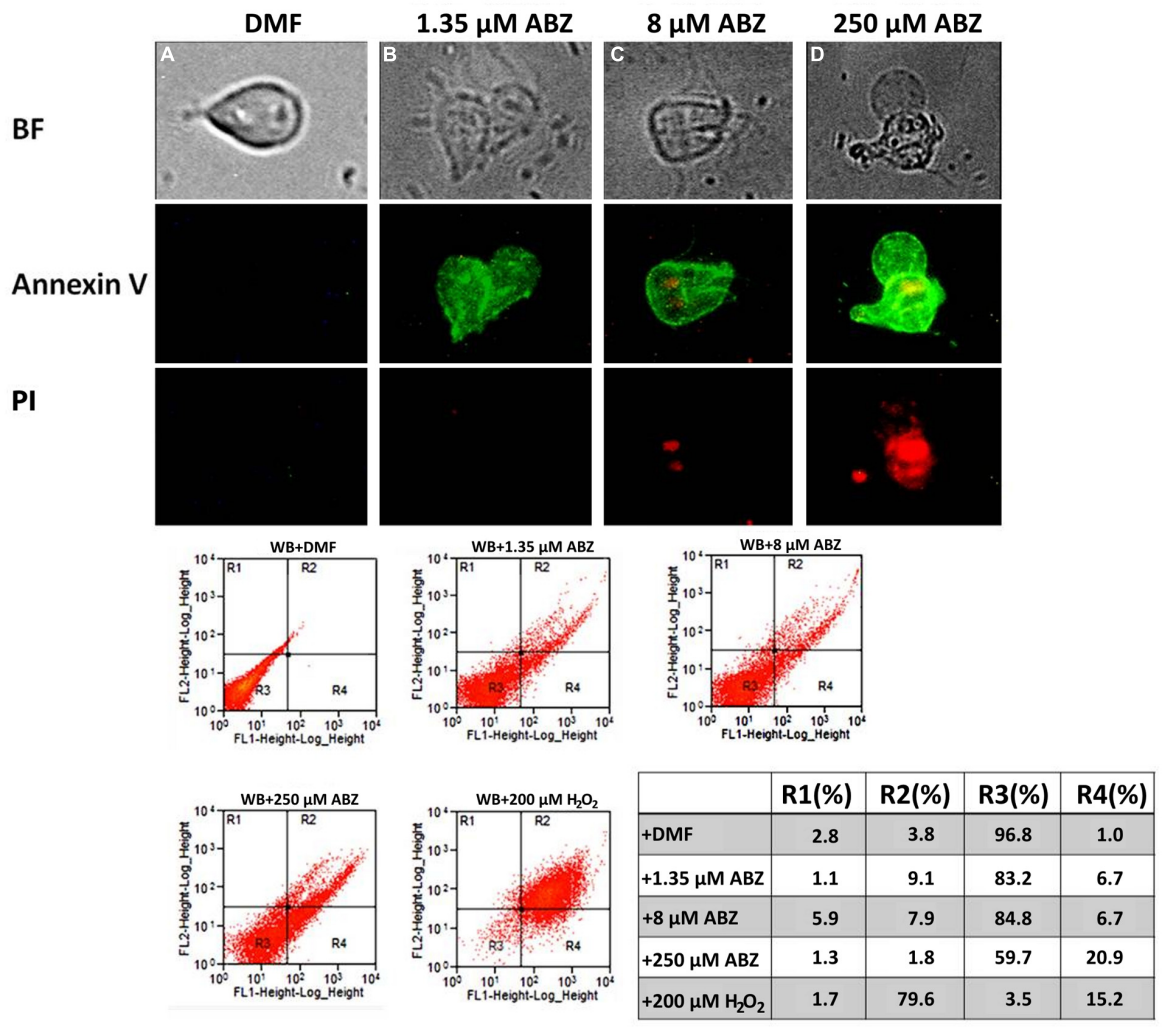
**Albendazole induces apoptosis-like death in *Giardia*.** Annexin V binding in the parasite’s cell surface (as indicator of early apoptosis) was detected as a consequence of translocation of phosphatidylserine to the plasmatic membrane (middle panel). The parasites were exposed to DMF **(A)**, 1.35 μM **(B)**, 8 μM **(C)**, 250 μM **(D)** of ABZ for 16 h at 37°C. Then annexin V-FITC and PI (indicator of late apoptosis and necrosis) were added to the medium. Trophozoites´ micrographs are as follows: top panel: in BF; bottom panel: trophozoites stained with annexin V-FITC and PI. The FITC and PI fluorescence was determined using a FACS Calibur Flow Cytometer. Data shown in the figure are as follows: quadrant R1 are cells positive for necrosis; quadrant R2 are cells positive for necrosis and apoptosis; quadrant R3 are cells negative for both markers; quadrant R4 are cells positive for early apoptosis. As control, flow cytometry was performed with WB cells exposed to 200 μM H_2_O_2._ The figures are representative of at least three independent experiments. The table shows population percent in each quadrant according to flow cytometry data.

**FIGURE 8:**
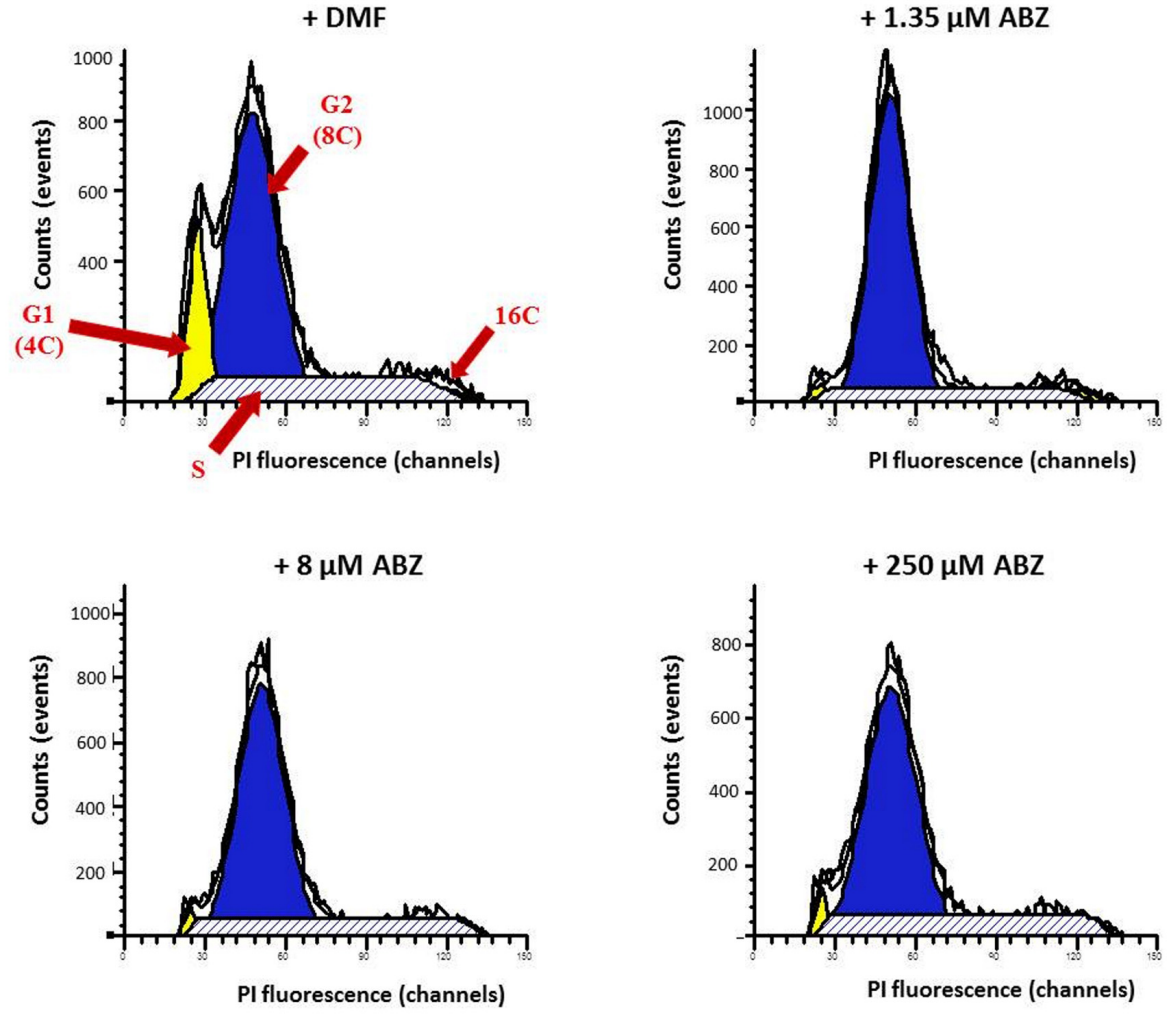
**Cell cycle stages determined by flow cytometry analysis in *G. duodenalis* trophozoites exposed to ABZ.** Parasites were exposed to DMF (control) and to different ABZ concentrations (1.35, 8, and 250 μM) for 4 h at 37∘C. Then cells were stained with propidium iodide (PI) and PI fluorescence was measured using a FACS Calibur Flow Cytometer and fitted with a FL-2 filter (585 nm). Cells in the G1 (yellow area), G2 (blue area), S (stripped area), and M (indicated as 16C) cell cycle phases are as indicated with red arrows. The figures are representative of at least three independent experiments.

To determine if ABZ causes necrosis or an apoptotic-like phenomenon, the translocation of phosphatidylserine in ABZ treated trophozoites was detected using anti-annexin *V* antibodies as a specific marker of early apoptosis. Trophozoites exposed to ABZ displayed a significant increase in annexin *V* staining. At higher drug concentration a significant number of cells positive to both markers indicated a process of cell death involving late apoptosis and necrosis (**Figure 7**). In further experiments the trophozoites nuclei were stained with PI to assess if ABZ exposure may alter the cell cycle progression between control and ABZ-exposed trophozoites. As can be observed in **Figure 8**, ABZ produced a noticeable and consistent decrease in the G1 subpopulation (yellow area, from 14.6% in vehicle-treated cells to 2.7% in 250 μM ABZ-treated cells), a slight decrease in the *S*-phase subpopulation in 1.35 μM ABZ-treated cells as compared to DMF-treated cells (stripped area, from 27 to 22.1%, respectively) and a moderated increase in G2 subpopulation between these same samples (blue area, from 61.0 to 69.9%, respectively). The cells treated with 8 μM ABZ displayed a similar distribution to the one observed in the population exposed to 250 μM ABZ. These data suggest that cytotoxic ABZ concentrations allow only a partial G1→S→G2 transit in cells in a pattern (G2 >> S > G1 > M) that indicates an arrested state at the G2/M phase boundary.

## Discussion

Parasitic diseases by protozoa and helminths represent a serious health problem worldwide. The control of the infections caused by these parasites is carried out mainly by drug treatment. The drug ABZ is a broad spectrum benzimidazole with an anti-helminthic effect and its low cost makes this drug a suitable candidate for mass drug administration programs to deworm children in countries where giardiasis is endemic as well. However, up to five 400 mg doses are needed to clear infections by *G. duodenalis*, limiting the utility of these campaigns to diminish burdens of giardiasis. Also, the use of suboptimal doses may engender and disseminate ABZ-resistant *Giardia* (Tian et al., 2010; Watkins and Eckmann, 2014). Likewise many parasites including *Giardia* exhibit cross-resistance to diverse drugs. Therefore, it is essential to understand the mechanisms involved in the toxicity of the drugs used; this understanding can help to devise high efficiency regimes and new and effective drugs against resistant strains (Watkins and Eckmann, 2014). Understanding the mechanisms of the effects of ABZ on *Giardia* will also help to define at least in part, the ABZ toxicity reported in some patients particularly in the liver, as well as other side effects such as possible teratogenisis (Dayan, 2003; Nandi and Sarkar, 2013).

In this context, the mechanisms of ABZ action have been studied mainly in helminthic parasites and information in protozoans is limited. The main mechanism of ABZ action involves binding to β-tubulin causing the destabilization of the cytoskeleton. In this regard, resistance to ABZ has been associated with point mutations in this protein and so far other effects of ABZ have received little attention. Some studies in mammalian cells have correlated the use of ABZ to the presence of oxidative stress. Since the susceptibility of many parasites to oxidative stress has been reported (Pal and Bandyopadhyay, 2012), in this study we have evaluated the capacity of ABZ to induce oxidative stress in the protozoan parasite *G. duodenalis*.

The results indicated that ABZ exposure induced intracellular ROS formation in drug susceptible trophozoites and interestingly ROS were detected mainly in the nuclei. Therefore, oxidative-stress caused by ABZ can be part of the cytotoxic effect of this drug in *Giardia.* An important aspect to be addressed in future studies is to determine which ROS are involved as well as the mechanisms inducing their formation.

Since ABZ cytotoxicity was herein related to oxidative stress, another pro-oxidant molecule (H_2_O_2_) was evaluated. Of note, a partial degree of cross-resistance to this agent was detected in the ABZ-resistant clones suggesting that similar antioxidant responses may be induced by each compound. This correlation between drug resistance and antioxidant response has also been suggested in drug resistant *Leishmania* strains (Berg et al., 2015) and in other pathogens such as *Plasmodium* and *Pseudomonas* (Lehane et al., 2012; Poole, 2014). The phenomenon of cross-resistance in *Giardia* to different drugs including metronidazole and ABZ has already been reported (Müller et al., 2007) although the causes of this have not yet been fully elucidated. The protection conferred by cysteine in ABZ-treated trophozoites further suggests that this drug induces some toxicity through oxidative stress thus cysteine may provide a reductive environment, however, the mechanisms involved in this protective effect remains to be elucidated. Interestingly in a recent study of our group, a set of antioxidant enzymes present in *Giardia* (e.g., NADH oxidase, flavoprotein-A, superoxide reductase, peroxiredoxins, Li and Wang, 2006; Mastronicola et al., 2014) have shown an increased expression along to increased intracellular cysteine concentrations in the ABZ-resistant clones used in this study (Argüello-García et al., 2015). Other processes as drug metabolization could also play a role in resistance because *Giardia* apparently has metronidazole-activating enzymes (nitro reductases; Pal et al., 2009) and ABZ metabolites (sulphoxide and sulphone) have been detected in this parasite (Oxberry et al., 2000). Interestingly these are accumulated in lower levels in the ABZ-resistant clones (Argüello-García et al., 2015).

Regarding the damage promoted by ABZ in trophozoites, two interesting findings were observed: (a), the lack of lipid peroxidation and protein carbonylation, and (b) the presence of DNA damage as a main mechanism of the cytotoxic effect of ABZ. The lack of lipid peroxidation may be due either to the fact that *Giardia* uptakes lipids and cholesterol from exogenous sources, to the unique lipid metabolism in this parasite, or to the nuclear localization of ROS that may limit damage to membrane-associated lipid metabolites (Gibson et al., 1999; Das et al., 2002). Moreover, protein carbonylation, an irreversible oxidative damage, showed only a tendency (not statistically significant) to increase when high ABZ concentrations were used, despite being easily detected when the parasites were treated with H_2_O_2_. Although carbonylation is an important marker for protein damage, it will also be important to analyze if other mechanisms of oxidative damage to proteins (Møller et al., 2011) may occur in the ABZ treated trophozoites.

The detection of 8OHdG adducts, together with DNA degradation in ABZ exposed trophozoites indicated that the DNA was the most affected molecule by the pro-oxidant action of ABZ. The *in vitro* damage of DNA by drugs such as metronidazole has been recently shown in *Giardia* (Uzlikova and Nohynkova, 2015). Other redox active drugs such as benzinidsazole and hydroxymethylnitrofurazone have been reported to affect mainly DNA (Davies et al., 2014), a pattern commonly present in necrosis. Further evidence of DNA damage in ABZ-treated trophozoites included histone H2AX phosphorylation that indicates a repair signal after DNA double strand break. In particular, H2AX phosphorylation observed in parasites treated with 250 μM ABZ could be due to the cell damage induced by the drug. Thus, phosphorylation of histone H2AX will be activated upon damage of trophozoites by ABZ. As shown in **Figure 7**, a process of necrosis appears particularly significantly when the parasites are exposed to 250 μM and in this condition H2AX phosphorylation occurs.

In other parasites such as *Toxoplasma gondii*, the phosphorylation of H2AX has been correlated with loss of pathological potential and reduced growth rates (Vonlaufen et al., 2010). It is worth mentioning that the typical ladder pattern of DNA degradation observed in *Giardia* exposed to ABZ was not entirely similar to the one associated with apoptosis in other organisms or as commonly present in necrosis (Bagchi et al., 2012). Thus the DNA degradation pattern correlates with an apoptotic-like phenomenon present in *G. duodenalis.* Indeed, the detection of phosphatidylserine translocation to the outer side of the cell membrane suggested an apoptotic-like event.

Our findings resemble studies reported by others regarding an apoptotic-like process in *Giardia* when it is exposed to classical inducers of oxidative stress and to metronidazole (Bagchi et al., 2012). Interestingly a partial arrest in S and G2 phases of the cell cycle was observed in ABZ-exposed trophozoites that correlate with the induction of oxidative stress and an apoptotic-like process. It is noteworthy that the cell cycle arrest is not complete and this allows a portion of the cell population to continue through the cell cycle. The effect of different drugs on the *Giardia* cell cycle has been reported to show different phenomena, including partial arrest in different cell cycle stages depending on the drug under study (Reaume et al., 2013). In our work, ABZ-treated trophozoites showed a pattern consistent with *Giardia* trophozoites displaying incomplete cytokinesis upon exposure to microtubule-acting compounds (Mariante et al., 2005).

In summary, this work demonstrated that ABZ induces the formation of intracellular of ROS in *G. duodenalis* leading to an oxidative stress status where the main affected biomolecule is DNA. This damage involves the formation of 8OHdG adducts and double-strand DNA break. This damage in turn leads to cell cycle dysregulation and eventually apoptotic-like cell death. These observations allow us to expand our understanding on the cytotoxic mechanism of ABZ in *Giardia* and opens future directions to a rational drug design for giardiasis, considering antioxidant responses as likely mechanisms of multidrug resistance. In this context, generating ROS or inhibiting endogenous antioxidant enzymes would be a rational approach to developing new anti-*Giardia* drugs as previously proposed for other parasites (Pal and Bandyopadhyay, 2012).

## Conflict of Interest Statement

The authors declare that the research was conducted in the absence of any commercial or financial relationships that could be construed as a potential conflict of interest.
